# The Prevalence of Dental Caries Among Children Aged 6–11: A Cross-Sectional Study from Mureș County, Romania

**DOI:** 10.3390/medicina61091648

**Published:** 2025-09-11

**Authors:** Ana-Gabriela Seni, Andreea Sălcudean, Ramona Amina Popovici, Iustin Olariu, Mădălina-Gabriela Cincu, Viorel Jinga, Laria-Maria Trusculescu, Dana Emanuela Pitic, Raluca Mioara Cosoroabă, Andreea Kis, Cristina Ioana Talpos-Niculescu, Liana Todor, Monica Tarcea

**Affiliations:** 1 Doctoral School of Faculty of Medicine, “George Emil Palade” University of Medicine, Pharmacy, Science and Technology from Târgu Mureș, 540139 Târgu Mureș, Romania; gabriela.seni@umfst.ro; 2 Department of Ethics and Social Sciences, George Emil Palade University of Medicine, Pharmacy, Science and Technology of Târgu Mureș, 540142 Târgu Mureș, Romania; 3 Department of Management and Communication in Dental Medicine, Faculty of Dental Medicine, Victor Babes University of Medicine and Pharmacy of Timișoara, 300041 Timișoara, Romania; 4 Department of Dentistry, Faculty of Dentistry, “Vasile Goldiș” Western University of Arad, 310025 Arad, Romania; olariu.iustin@uvvg.ro; 5 Faculty of Medicine, George Emil Palade University of Medicine, Pharmacy, Science and Technology of Târgu Mures, 540142 Târgu Mureș, Romania; cincu.madalina@yahoo.com; 6 Department of Urology, University of Medicine and Pharmacy “Carol Davila” Bucharest, 020021 Bucharest, Romania; viorel.jinga@umfcd.ro; 7 Faculty of Dental Medicine, Victor Babes University of Medicine and Pharmacy of Timisoara, Eftimie Murgu Sq., 300041 Timisoara, Romania; laria.trusculescu@umft.ro (L.-M.T.); dana.emanuela@gmail.com (D.E.P.); cosoroaba.raluca@umft.ro (R.M.C.); ioana.talpos-niculescu@umft.ro (C.I.T.-N.); 8 Research Center for Pharmaco-Toxicological Evaluations, Faculty of Pharmacy, “Victor Babes” University of Medicine and Pharmacy, Eftimie Murgu Sq., No. 2, 300041 Timisoara, Romania; kis.andreea@umft.ro; 9 Department of Dental Medicine, Faculty of Medicine and Pharmacy, University of Oradea, 10 Decembrie Sq., 410068 Oradea, Romania; liana.todor@gmail.com; 10 Department of Community Nutrition, Faculty of Medicine, “George Emil Palade” University of Medicine, Pharmacy, Science and Technology from Târgu Mures, 540142 Târgu Mures, Romania; monica.tarcea@umfst.ro

**Keywords:** dental caries, schoolchildren, risk factors, oral hygiene, toothbrushing

## Abstract

*Background/Objectives*: The prevalence of dental caries in Romania is significantly high, especially among children and adolescents. We aimed to assess the prevalence of dental caries and their associated factors among schoolchildren aged 6–11 years learning at urban and rural schools from Mureş County, Romania. *Methods*: This cross-sectional study included a sample of 1124 children, aged 6–8 years (*n* = 524), as well as aged 9–11 years (*n* = 600). Nine schools in Mureş County, Romania, were selected for screening, based on their location (4 schools from urban areas and 5 schools from rural areas). Data were collected based on children’s visual dental screenings and a self-administered questionnaire addressed to their parents to collect information about oral health behaviors, sugar consumption, and dental care history. Dental clinical examination was performed by specialists, and DMFT/dmft values were recorded. Binary logistic and negative binomial regression analyses were used to assess the factors associated with dental caries. *Results*: Among 6–8-year-olds, the prevalence of untreated decay was 76.5% and the prevalence of caries experience was 77.7% (mean dmft = 3.9). Among 9–11-year-olds, the prevalence of untreated decay was 43.5% and the prevalence of caries experience was 48.2% (mean DMFT = 1.9). Among the most significant factors associated with caries prevalence were school location (*p* = 0.04 for children aged 6–8 years, and *p* < 0.001 for 9–11 years); the employment status of mothers (*p* = 0.04 for 9–11 years); eating sweets ≥4 times/day (*p* = 0.04 for 6–8 years); brushing time ≥3 min (*p* = 0.03 for 9–11 years); as well as past dental restorative treatments or emergency (*p* < 0.001 for all the children examined). *Conclusions*: Preventive measures and innovative educational interventions are needed to mitigate the impact of dental caries prevalence on the health and education of schoolchildren.

## 1. Introduction

Dental caries still represents a significant public health problem, especially among children. A wide range of factors, including biological factors, socioeconomic status, dietary habits, and oral hygiene practices, influence the development of dental caries among the pediatric population [1,2]. Biological factors influencing caries development include saliva and microbiota. Saliva is vital for oral health, as it offers protective mechanisms against caries. The microbial composition of saliva is essential for understanding dental caries etiology. Elevated levels of *Streptococcus mutans* are linked to caries development. Studies show that children with caries have more bacteria in their dental plaque than those without [3,4,5]. Recent research suggests a complex interaction of various microbial species in caries, which includes *Lactobacillus* strains and fungi like *Candida*, particularly in severe early childhood caries [6,7,8]. Low socioeconomic status increases the risk of dental caries in children due to limited access to dental care and lower levels of parental education and health literacy. Limited access to preventive care and oral hygiene education leads to a higher incidence of caries in these families [9,10,11]. The parental occupation and education significantly affect children’s oral health behaviors and access to dental services, contributing to disparities in caries prevalence [12]. Diet significantly affects the development of dental caries in children, with high sugar intake, especially free sugars, identified as a crucial risk factor. Increased sugar consumption is associated with elevated levels of cariogenic bacteria [13,14]; therefore, the children are more prone to dental caries, underscoring the necessity for dietary interventions in conjunction with traditional oral health education [15]. Effective oral hygiene is crucial for lowering dental caries risk. Regular brushing at least twice daily, using fluoride toothpaste, mouthwash, and dental floss, is a vital preventive measure for children. Research indicates that good self-hygiene considerably decreases caries incidence, particularly on smooth tooth surfaces [16,17]. Additionally, fostering oral hygiene in children requires proactive parental involvement; children with knowledgeable parents are more likely to sustain effective oral hygiene practices [18,19].

Dental caries impacts children’s overall well-being and academic performance, as their prevalence correlates with poor school attendance and learning difficulties [20,21]. Additionally, children suffering from caries experience a diminished quality of life due to discomfort and impaired dental function [22]. Inadequate dental hygiene can lead to serious health issues, including nutritional deficiencies from pain related to caries, which can hinder children’s growth and development [23]. Thus, addressing dental caries is crucial not only for oral health but also for the broader implications on children’s well-being and development.

The World Health Organization (WHO) classifies dental caries as a significant health issue, affecting 60% to 90% of schoolchildren globally [24]. Studies indicate a high prevalence of dental caries in various populations of children [25,26,27,28,29,30,31,32]. A longitudinal study in Thailand revealed that dental caries prevalence increased from 34.2% at 24 months to 68.5% at 36 months of age [33]. Consequently, children with a history of dental caries are at greater risk for developing new caries, highlighting the cyclical nature of this disease, where early onset heightens future risks [13,34].

In Romania, the prevalence of dental caries among children and adolescents represents a significant public health problem, characterized by a high prevalence, deeply linked to socioeconomic challenges and dietary behaviors. For example, a study conducted in the south-central regions of Romania found that approximately two-thirds of participants aged 12 years had some form of dental caries, in close correlation with socio-behavioral factors such as dietary habits and oral hygiene practices [35]. Furthermore, a fairly high prevalence of caries (95.5%) was also observed among adolescents aged between 10 and 19 years in Cluj-Napoca, Romania [36]. The DMFT (Decayed, Missing, Filled Teeth) rates for children in the two studies were particularly high, and align with national trends observed in other research [37,38]. Longitudinal data indicate that although there has been a slight improvement in caries prevalence among Romanian children, the DMFT index remains persistently higher compared to Western European countries [39]. Specifically, the DMFT index for 12-year-olds in Romania was documented at 3.8 in 1995, down from 5 in 1985, but this figure contrasts sharply with a DMFT score of 0.4 in Denmark (in 2014), 0.5 in Germany (in 2014), 0.7 in Spain (in 2014) and the United Kingdom (in 2011), or 0.8 in Sweden (in 2011) [40,41], compared to 3.13 in Romania (in 2020) [36]. More recent studies have corroborated this trend, reporting a dmft of approximately 4.89 in 6–8-year-old children, suggesting a prevalence of dental caries of approximately 75% within this demographic group [42], and a DMFT of 2.93 for children with a mean age of 12 [43]. This trend observed can be attributed to a correlation between low incomes, low education levels, and the increased prevalence of dental caries, particularly affecting rural populations [42,44]. An observational study conducted in 2019 indicated that the prevalence of tooth decay in Romania was up to 96.3% among young people and 97.5% among adults, revealing the ubiquitous nature of this condition [45]. Therefore, there is a need to reshape oral health policies in Romania, as highlighted by Cernega and co-workers [46]. Although the number of dentists has increased considerably in Romania, especially in the private sector where the fees charged put a great financial pressure on patients, the insufficient financing of dental procedures by the Romanian Ministry of Health (MoH), the reduction of dental procedures reimbursed by the National Health Insurance Fund (NHIF), and the limited number of dentists who have concluded contracts with NHIF significantly contribute to a stagnation or even a decline in the oral health outcomes of Romanian patients [46].

In the context of the above, we aimed to investigate the prevalence of dental caries and the factors associated with their occurrence among children aged 6 to 11 years who study in both urban and rural schools in Mureş County, Romania. In the study, both socioeconomic factors and practices related to children’s dental hygiene (frequency and time of tooth brushing, dental consult) were investigated. Furthermore, the frequency and consumption of sugary products (sweets, carbonated juices, and sweet fruit juices) were investigated to determine the association of these products with the occurrence of caries. Mureş County is situated in the central part of Romania, and is composed of 102 administrative-territorial units, comprising 4 municipalities (among which Târgu Mureș is the county seat), 7 cities, and 91 communes. According to the national census in 2022, the population of Mureş County was 518,193 inhabitants. In 2021, the average net salary in Mureş County was 3117 RON (623 euros). Regards the distribution of population by place of residence, in Mureş County, 246,721 people live in urban areas, representing 47.61% of the Mureş County population, while 271,472 people live in rural areas (52.39%) [47,48,49].

We believe this research is highly significant because it highlights the high prevalence of dental caries among children in Romania. Despite an increase in dental services, there are still disparities in oral care resulting from socioeconomic challenges and limited funding in the healthcare system. This study focuses on urban and rural populations in Mureș County, exploring a wide range of factors contributing to caries prevalence (socioeconomic status, oral hygiene practices, habits, and access to dental care), which makes it innovative. Therefore, this research highlights the cyclical nature of dental caries and its broader impact on quality of life, school performance, and child growth and development. This research aims to fill the gaps in localized data by correlating behavioral, socioeconomic, and dietary factors specific to this region, thus providing specific information needed for the development of effective and culturally sensitive public health policies. Given the global burden of dental caries and its known links to socioeconomic inequality and dietary behaviors, the contextual approach of this study makes it a vital contribution to preventive strategies adapted to Romania.

## 2. Materials and Methods

### 2.1. Study Design and Participants

An oral health survey with sugar consumption of random samples of schoolchildren aged 6–11 years old from schools in Mureş County, Romania was performed. In this descriptive cross-sectional study, data collected from 1124 participants comprising the group of schoolchildren aged 6–8 years, *n* = 524, and the group of schoolchildren aged 9–11 years, *n* = 600, from Mureş County, Romania, during 4 months (March–June 2025) were used. The study followed the international standards established by the World Health Organization (WHO) [50]. The sample included 9 primary schools from Mureş County, 4 schools from the urban area, and 5 schools from the rural area.

The inclusion criteria were based on the children’s age; in the first group, children who had reached their 6th birthday but were not yet 9 years old were included, and in the second group, children who had reached their 9th birthday but were not yet 12 years old were included. The exclusion criteria were based on mental health or physical disease (e.g., intellectual disability, muscular paralysis, cerebral palsy, autism spectrum disorders, central nervous system disorders, including those caused by perinatal hypoxia, chronic diseases, reduced saliva secretion (xerostomia), etc.); children who suffer from these diseases were excluded to avoid potential influences of associated diseases. The children were selected based on their dentition. The study sample included children aged 6 to 11 years, with detailed age-specific analyses performed at the internationally recognized index ages of 6, 8, and 11 years. These ages correspond to peak periods of deciduous (6 and 8 years) and early permanent dentition (11 years), in line with WHO and national monitoring guidelines.

### 2.2. Ethical Consideration

The study protocol was approved by the Scientific Research Ethics Committee of the “George Emil Palade” University of Medicine, Pharmacy, Science and Technology from Târgu Mureş (no. 3202 of 3 June 2024), as well as by the School Board of each school in urban and rural areas in Mureş County. The participation of the children was voluntary, and they were subsequently screened based on the study’s inclusion criteria. Parents were provided with information regarding the content and conduct of the study, as well as the written informed consent in the Romanian language, which was obtained from all parents before their children were dental screened. Moreover, before the dental clinical screening, to ensure the granting of consent, the child’s parents were invited to complete a survey, which included questions regarding the basic socioeconomic characteristics, as well as questions about their child’s oral health habits, tooth brushing habits, sugar intake, and dental consult habits. The questionnaires were self-administered, and the class teachers were available to provide clarification regarding the questions, to encourage honest and complete responses from parents, based on the relationship between them. Each survey completed and each child examined was recorded under a unique code to ensure data confidentiality.

### 2.3. Data Collection

Two working teams, comprising a dentist and a hygienist, performed the children’s dental clinical examination and collected data from the survey. The children’s dental clinical examination consisted of the number of healthy teeth, the number of carious teeth, the teeth with fillings, and the missing teeth due to decay. The dental screening consisted only of a visual evaluation, and the specialists used a dental mobile office equipped with dental mirrors, tongue depressors, reflector lights, as well as gauze and toothpicks to clean children’s teeth of food remnants. The mixed dentition status was not followed, only the presence of primary teeth (for the first group of participants) and the presence of permanent teeth (for the second group of participants). The oral hygienist, together with the class teacher, distributed the questionnaires, which included, among the demographic factors, the children’s gender, school location (urban or rural), parents’ education level (high school or university studies), and employment status.

To ensure consistency and reproducibility of clinical caries assessments, all examiners underwent a calibration process before the onset of data collection. The calibration procedure included a two-day training session based on the World Health Organization diagnostic criteria for dental caries. During this session, both theoretical definitions and practical clinical exercises were conducted. For reliability testing, a subsample of 30 children, representing both age groups, was independently examined by all participating examiners. The presence/absence of untreated caries and the number of affected teeth were recorded. Inter-rater reliability was evaluated using Cohen’s kappa coefficient for categorical outcomes (presence of dental caries) and the intraclass correlation coefficient (ICC) for continuous outcomes (number of decayed teeth).

During the actual fieldwork, each child was examined by one calibrated examiner; children were not re-examined by both teams to minimize discomfort and disruption to the school schedule. However, examiners periodically cross-checked diagnostic consistency by jointly reviewing randomly selected cases throughout the study. These procedures ensured that outcome variables were assessed with a high degree of reliability and methodological rigor.

As regards the sugar intake, the questions consisted of consumption of sweets, soda, or natural juice, ranging from never or rarely to ≥4 times/day. As regards the oral healthy habits, the questions consisted of frequency of toothbrushing, ranging from never or rarely to ≥4 times/day; the brushing time, ranging from under 1 min to ≥3 min; and the dental consult, ranging from routine consult, restorative treatment, or in case of emergency. The parents were asked to mention the reason for missing a dental consult, such as financial, lack of knowledge of the best dentist, lack of time, lack of trust in dentists, or other reasons specified by parents, in the case of children who had never visited a dental clinic.

### 2.4. Variables Followed in the Study

We distinguished between untreated caries and overall caries experience:

1. Untreated caries (dt/DT): the number of teeth with untreated decay (primary dentition: dt; permanent dentition: DT). We derived two outcomes: (a) a binary prevalence of untreated decay (coded 1 if dt > 0 or DT > 0; 0 otherwise) and (b) a count outcome (the number of untreated decayed teeth: dt for ages 6–8; DT for ages 9–11).
2. Caries experience (dmft/DMFT): the sum of decayed (treated or untreated), missing due to caries, and filled teeth (primary dentition: dmft; permanent dentition: DMFT). We report descriptively the prevalence of caries experience (dmft > 0/DMFT > 0), the mean caries experience (mean dmft/DMFT), and the Significant Caries Index (SiC).

The Significant Caries Index (SiC) was computed as the mean dmft/DMFT of one-third of the sample with the highest dmft/DMFT values, following WHO recommendations [51].

Independent variables (covariates): Based on the literature and data availability, we considered multiple categories of predictors: sociodemographic factors (age group, gender, school location (urban/rural), parents’ education level, and parents’ employment status); behavioral factors (frequency and duration of tooth brushing, prior dental visits, history of dental emergency or treatment); dietary factors (frequency of consumption of sweets, sugar-sweetened beverages, and natural fruit juice).

All variables were derived from a parental questionnaire. Variables with significant bivariate associations (*p* ≤ 0.05) were further analyzed in multivariable models.

To assess diagnostic consistency, inter-rater reliability was evaluated on a random subsample of children examined by both calibrated dentists. Cohen’s kappa coefficient for the prevalence of untreated caries (dt/DT > 0) was κ = 0.87, and the intraclass correlation coefficient (ICC) for the number of untreated decayed teeth (dt/DT count) was ICC = 0.91. Both values exceeded the accepted threshold of 0.80, demonstrating excellent agreement between examiners.

### 2.5. Statistical Analysis

Data analysis was performed by using the IBM-SPSS Statistics software platform (from IBM^®^ SPSS^®^ Statistics Corp. (Chicago, IL, USA), software version 24.0). Descriptive statistics were computed for all variables. Bivariate associations between predictors and caries outcomes were tested using Chi-square or *t*-tests as appropriate. The multivariable models were constructed for each of the two main outcome variables:

- Logistic regression estimated associations with the presence of untreated caries (binary outcome: dt > 0 or DT > 0 vs. 0).
- Negative binomial regression modeled the severity of untreated caries (count outcome: number of untreated decayed teeth; dt for ages 6–8 and DT for ages 9–11), accounting for overdispersion.

In addition, dmft/DMFT were summarized descriptively (means, SiC, and the proportion with dmft/DMFT > 0) to characterize overall caries experience.

Independent variables included in the multivariable models were:

- School location (urban/rural);
- Parental education;
- Parental employment status;
- Oral hygiene behaviors (tooth brushing frequency and duration);
- Dietary habits (consumption frequency of sweets, soda, natural fruit juice);
- Dental care history (past dental visit, treatment, or emergency).

Interaction terms between age group and each predictor were tested to assess moderation. Where significant, we stratified the models by age group (6–8 years and 9–11 years) and presented results separately.

In the multivariable models, all the socioeconomic and behavioral variables were included to minimize the risk of systemic biases in the statistical results (odds ratios (ORs), rate ratios (RR), *p*-values, and confidence intervals (CIs) of 95%) [52]. The *p*-value was set at ≤0.05.

As concerns the covariate selection, all covariates included in the multivariable logistic and negative binomial regression models were selected based on their theoretical and empirical relevance to the development of dental caries, as supported by previous research and guidelines from the WHO for oral health surveys [50]. Specifically, we considered sociodemographic variables (e.g., child’s gender, school location, parents’ education, and employment status), behavioral factors (e.g., frequency and duration of toothbrushing, dental visits), and dietary habits (e.g., frequency of consuming sweets, soda, and natural fruit juice). These variables were chosen a priori as potential explanatory or confounding factors in line with established literature. The analysis included all variables collected via the parental questionnaire, as we aimed to explore the multidimensional relationship between socioeconomic, behavioral, and dietary influences on caries prevalence and severity. Given the large number of statistical tests conducted across both unadjusted and adjusted regression models, we acknowledge the potential for inflated type I error rates due to multiple comparisons. However, the primary objective of this study was exploratory, aiming to identify and describe potential associations between a range of socioeconomic, behavioral, and dietary variables and the prevalence of untreated decay/caries experience among school-aged children in Mureș County, Romania. Accordingly, we chose not to apply formal corrections (e.g., Bonferroni, Benjamini–Hochberg false discovery rate) for multiple comparisons, as these can be overly conservative in exploratory public health research and may obscure meaningful associations that merit further investigation. Instead, we focused on presenting both effect sizes (ORs and RR) and CIs, alongside *p*-values, to assess the strength and precision of the observed associations.

We emphasize that statistically significant findings should be interpreted as hypothesis-generating rather than confirmatory, and that replication in future studies is warranted to validate the robustness of these associations. This approach aligns with recommended practices in epidemiological studies where comprehensive modeling is used to inform future targeted research.

## 3. Results

Among the 524 children aged 6–8 years evaluated, the proportion of boys and girls was approximately the same (49.2% vs. 50.8%), as well as their primary school location (urban vs. rural). One can observe that most parents of children aged 6–8 years old had university studies (mothers’ 67.9% vs. fathers’ 60.7%). As regards the employment status, most of the mothers were unemployed (52.5%) as compared with the unemployed fathers (37.2%).

Among the 600 children aged 9–11 years evaluated, the proportion of girls was a little higher (55.2%) compared to the boys’ proportion (44.8%), and most of the children studied in a primary school from a rural area (54%), as compared with 46% of children who studied in schools from the urban area. As in the previous group of children, most parents had a higher education level (69.8% of mothers and 65.2% of fathers), but 51.7% of mothers were unemployed, compared with 36.5% of unemployed fathers (Table 1).

Among the children included in the first group (6–8-year-olds), 77.7% had any caries experience; mean dmft was 3.9 ± 3.5, and the mean of the SiC score was 7.7 ± 2.5. Most of the children from the first group had untreated decay (76.5%), 6.3% of children had fillings, and 5.3% had missing teeth due to the decay. Although small differences between urban and rural schools were observed in the percentages of children with missing or filled teeth, these variations were minimal and are likely due to random sample variation (Table 2).

In the second group of children (9–11 years), it was shown that 48.2% had any caries experience; mean DMFT was 1.9 ± 1.8, and the mean SiC score was 3.3 ± 3.8. Most of the children in this group (43.5%) had untreated dental decay, 7.8% of children had missing teeth, and 6.3% of children had filled teeth. Concerning the schools’ location where children learn, small differences were observed between rural and urban schools in the proportion of children with missing or filled teeth (7.1% vs. 6.5% for missing teeth, and 7.1% vs. 5.4% for filled teeth). However, these variations are minimal and may be explained by random sample variation, as the adjusted analysis and mean dmft/DMFT values indicate no disadvantage for rural children (Table 2).

When the parents were asked about the reason they do not take their children to the dentist regularly, for the first group of children (6–8 years), the reason was as follows: the child does not need to visit the dentist (*n* = 402, 76.7%) (as considered by the parents, because the children do not complain of pain or discomfort), the parents had no time (*n* = 69, 13.2%), financial reasons (*n* = 18, 3.4%), lack of knowledge of the best dentist (*n* = 15, 2.9%), fear of the dentist (*n* = 12, 2.3%), or other reasons such as lack of trust in dentists (*n* = 8, 1.5%). For the second group of screened children, similar patterns were obtained.

### The Socioeconomic and Oral Health Behavior Factors Associated with Dental Caries

Table 3 depicts the children’s distribution related to sugar consumption and oral care habits. One can observe that regarding the children aged 6–8 years, eating sweets was the first choice among the responses, followed by drinking natural fruit juice and soda. Children aged 9–11 years prefer soda, followed by sweets and natural juice. As regards the past dental consult, about half of children visit dental offices when they have an emergency.

As concerns the prevalence of untreated decay (dt/DT), among children aged 6–8 years old, it can be observed that after adjusted odds ratio (OR), the most significant factors associated with the untreated decayed teeth were school location (OR = 0.53, CI 95% = 0.31–0.92, *p* = 0.04); eating sweets ≥4 times/day (OR = 1.13, CI 95% = 0.33–3.93, *p* = 0.04); past dental restorative treatments (OR = 3.83, CI 95% = 1.84–7.97, *p* < 0.001); and past dental emergency (OR = 9.93, CI 95% = 3.49–25.3, *p* < 0.001). Among children from the second group of screening, aged 9–11 years old, the most significant factors after adjusted OR, associated with the untreated decayed teeth, were also school location (urban/rural) (OR = 0.50, CI 95% = 0.34–0.75, *p* < 0.001); mother employment (OR = 0.64, CI 95% = 0.45–0.90, *p* = 0.04); brushing time ≥3 min (OR = 0.41, CI 95% = 0.21–0.81, *p* = 0.03); as well as past dental restorative treatments (OR = 2.25, CI 95% = 1.51–3.35, *p* < 0.001) and past dental emergency (OR = 2.78, CI 95% = 1.77–4.36, *p* < 0.001) (Table 4).

Concerning the dental caries experience (dmft/DMFT), presented in Table 5, one can observe that among the children from the first group of screening, aged 6–8 years old, the most significant factors associated with dental caries experience, after adjusted rate ratio (RR), were drinking natural juice ≥4 times/day (RR = 1.99, CI 95% = 1.05–3.81, *p* = 0.04); sweet consumption 2–3 times/day (RR = 1.81, CI 95% = 1.08–3.05, *p* = 0.04); as well as past dental consults for restorative treatment (RR = 1.93, CI 95% = 1.46–2.54, *p* < 0.001), or for emergency (RR = 2.03, CI 95% = 1.57–2.63, *p* < 0.001).

Concerning the children from the second group of screening, aged 9–11 years old, the results showed that the most significant factors associated with dental caries experience, after adjusted RR, were school location (RR = 0.61, CI 95% = 0.47–0.81, *p* < 0.001); mother employment (RR = 0.75, CI 95% = 0.59–0.95, *p* = 0.04); soda consumption several times/week (RR = 0.76, CI 95% = 0.57–1.0, *p* = 0.04); brushing time of 1 min (RR = 1.49, CI 95% = 1.07–2.07, *p* = 0.03); as well as the past dental consults, for restorative treatment (RR = 2.27, CI 95% = 1.72–3.01, *p* < 0.001), or emergency (RR = 2.63, CI 95% = 1.93–3.59, *p* < 0.001) (Table 5).

Table 6 depicts the dental health indicators according to sugar consumption, oral health behaviors, and dental consultations of children aged 6, 8, and 11 years regarding the caries outcomes, representing index ages for deciduous and permanent dentition. Prevalence is defined as the proportion of children with at least one untreated carious lesion. The dmft values were found to be higher in children aged 6 and 8 years who consume sweets and drink natural fruit juice, as compared to children who did not. Regarding the children aged 11 years, the DMFT value was found to be higher in children who drink soda than in children who eat sweets or drink natural juice.

Table 7 presents the SiC indices (± SD) for children aged 6, 8, and 11 years according to different dietary and oral health behaviour parameters. As expected, the SiC values were consistently higher than the corresponding mean dmft/DMFT scores reported in Table 6, reflecting the concentration of caries experience within the most severely affected third of children. Stratification by dietary factors showed that frequent sugar consumption was associated with markedly higher SiC values, particularly eating sweets at age 6 (SiC = 8.00 ± 2.22), drinking natural juice at age 8 (SiC = 8.55 ± 2.66), and consuming soft drinks at age 11 (SiC = 7.97 ± 9.14). Regarding oral health behaviors, the highest SiC values were observed among children reporting treatment or emergency dental visits (SiC = 10.62–12.44), indicating that those with insufficient preventive care are most likely to develop severe caries requiring professional intervention. These findings underline the unequal distribution of disease burden and support the evidence that behavioral factors, particularly sugar intake and irregular or reactive dental care, play a decisive role in caries outcomes (untreated decay and caries experience), in line with international observations [13,14].

**Figure 1:**
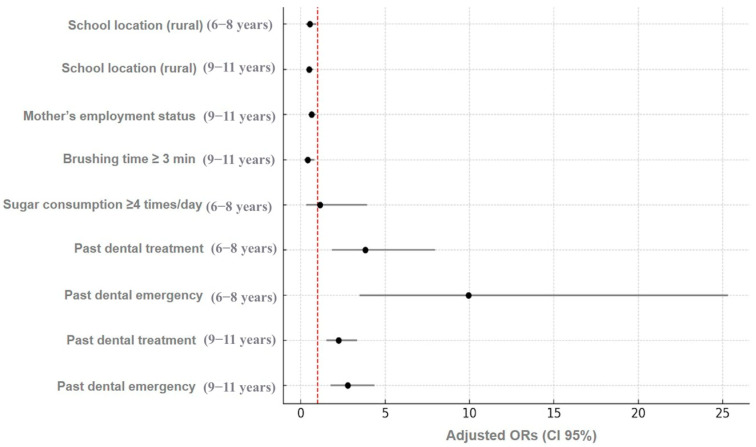
Forest plot of significant predictors of untreated decay. The forest plot summarizes the adjusted ORs and 95% CI for significant predictors of dental caries, stratified by age group (6−8 and 9−11 years). The vertical red dashed line at OR = 1 represents the threshold of no association. Predictors with CI that do not cross this line are considered statistically significant and suggest a meaningful association with the outcome.

Figure 1 provides a forest plot of adjusted odds ratios and 95% confidence intervals for significant predictors of untreated decayed teeth, stratified by age group. This visualization enables a concise and comparative overview of effect sizes and statistical significance across the most relevant variables.

**Figure 2:**
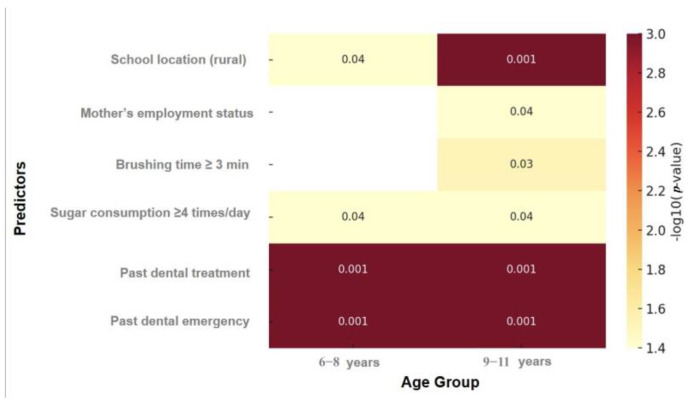
Heatmap displaying the level of statistical significance (*p*-values) for selected predictors of untreated dental caries, stratified by age group. Values are transformed to −log_10_(*p*) for visual clarity. Darker shades indicate higher statistical significance. White cells indicate missing or non-significant results.

Figure 2 presents a heatmap illustrating the level of statistical significance (*p*-values) for selected predictors of untreated dental caries, stratified by age group (6–8 and 9–11 years). The values are transformed to −log_10_(*p*) to enhance visual contrast, with darker shades indicating stronger statistical associations. This visualization enables rapid comparison of significance levels across predictors and age groups, highlighting variables such as past dental emergency and past dental treatment as consistently significant in both age groups.

**Figure 3:**
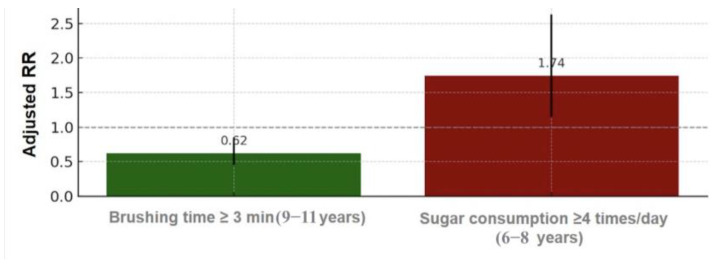
Grouped bar chart illustrating adjusted rate ratios (RR) with 95% CI for behavioral predictors of dental caries experience. Brushing for ≥3 min was associated with significantly lower caries experience (RR < 1), while frequent consumption of sweets (≥4 times/day) was associated with higher caries experience (RR > 1). The dashed line at RR = 1 represents the threshold for no association.

Figure 3 shows a grouped bar chart of adjusted rate ratios (RRs) with 95% CI for two key behavioral predictors of dental caries experience. Brushing for ≥3 min daily (among children aged 9–11 years) is associated with significantly lower caries experience (RR < 1), whereas high-frequency sweet consumption (≥4 times/day) in the children of the 6–8-year-old group is associated with increased caries experience (RR > 1). The chart emphasizes the opposing effects of protective versus risk-related behaviors on oral health outcomes.

## 4. Discussion

The results showed that three-quarters of the children aged 6–8 and half of the children aged 9–11 had already experienced caries in the primary or permanent dentition; in most of them, the cavities were not treated because the children were not used to visiting dental offices regularly for dental care or dental treatments. In our sample, untreated decay affected 76.5% (6–8 years) and 43.5% (9–11 years), while caries experience (dmft/DMFT > 0) was 77.7% and 48.2%, respectively (mean dmft = 3.9; mean DMFT = 1.9). The results obtained in the case of children from the first group evaluated correlate with the self-reported behavior related to dental care. According to other research studies, our results were lower (in the case of children aged 9–11 years), similar, or higher (in the case of children aged 6–8 years) regarding the prevalence of tooth decay reported for children from various regions. For example, a study conducted in Kathmandu, Nepal, reported a prevalence of dental caries of 55.84% among children aged 6 to 12 years, attributing this to poor oral hygiene practices and high consumption of sugary foods [26]. Similarly, research conducted in Madurai, India, indicated a prevalence of 43% among primary school children, which is consistent with various Indian studies reporting prevalence rates of up to 78.9% [27,28,53]. These figures highlight a worrying trend in which dental caries remains prevalent, particularly in developing countries where access to dental care is limited. The studies conducted among Syrian immigrant children aged 6–12 years revealed rates of up to 68.89% [29]. The same percentage was also reported by Podel and colleagues following their investigation of the prevalence of dental caries among school children of a private school in Bharatpur metropolitan city [25]. Additionally, a survey conducted in Dolakha, Nepal, indicated an increased prevalence of dental caries of 90.2% among children aged 3–15 years from both private and public schools [32]. A higher prevalence of dental caries of 78.8% was reported among schoolchildren aged 7 to 13 years in Kabul city [30], as well as a prevalence of 85.83% with a mean DMFT of 3.01 ± 2.24 among school children aged 3–14 years of Chitwan, with a higher prevalence in females compared to males [31].

Common factors contributing to their occurrence include poor eating habits, lack of regular dental check-ups, and insufficient education on oral health among both children and parents [27,54,55]. Educating children about dental care is essential in promoting oral health throughout their lives. Both the school and family environments are critical because they significantly influence children’s dental health behaviors and practices. Early and ongoing dental education can significantly improve children’s oral hygiene practices. The study reported by Pargaputri et al. demonstrates that educational interventions on dental and oral health lead to notable improvements in oral hygiene, including tooth brushing and caries prevention [56]. Parental involvement is particularly essential in these educational processes, as informed parents are more prepared to pass on dental health knowledge to their children. Furthermore, Olak and co-workers suggest that maternal education has a significant impact on children’s dental health, emphasizing the role of mothers as educators for health education [19]. School-based programs are vital in reducing oral health disparities among children from different socioeconomic backgrounds. These programs emerged in response to findings indicating that oral health status is often correlated with socioeconomic factors and serve as effective tools for mitigating inequalities related to parental education levels and for improving overall acceptance and utilization of dental health services in communities [57]. Lessons taught in schools can complement the education children receive at home, creating a comprehensive learning environment that promotes better dental health practices. In recent years, oral health curricula have begun to appear in Romanian schools, and programs adapted for schoolchildren have demonstrated the potential to improve oral health literacy, leading to improved dental practices at home [42].

In our study, the mean caries experience among 9–11-year-olds was 1.9 ± 1.8. The WHO global dental health target, however, is defined for 12-year-olds, with a benchmark DMFT ≤ 3.0. Therefore, our results are not directly comparable to the WHO target and should be interpreted with caution; the benchmark is reported here only for context and takes into account the fact that the children in our study, even if they had not turned 12 at the time of the study, were over 11 years old (some even 11 years and 10 months). We also calculated the SiC index to complement this measure [58]. This indicator reflects the distribution of caries burden within the population, highlighting the sub-group with the highest risk. The WHO initiative, initially proposed in 2000, aimed to reduce the prevalence of dental caries among children by 50%, with particular emphasis on achieving an overall average DMFT < 3.0 for 12-year-olds [59,60]. The Significant Caries Index (SiC), calculated as the mean DMFT of the one-third of children with the highest scores, was determined in accordance with WHO methodology, but it was not analyzed in relation to socio-demographic characteristics in this study. It has been reported that children from lower socioeconomic backgrounds often exhibit higher SiC scores, which may reflect inadequate access to oral health education and resources, strengthening the argument for tailored dental health initiatives that address specific community needs [61,62]. For example, in Libya, a mean DMFT score of 1.7 was reported among children aged 6–11 years, which is below the WHO target but indicates problems within certain socioeconomic sectors and class distributions, thus underlining the relevance of SiC assessments as complementary indicators of oral health [63].

By reporting caries outcomes separately for ages 6, 8, and 11 years, this study aligns with established epidemiological standards. These index ages are widely used in WHO oral health surveys and reflect developmental stages with distinct caries risk profiles. The highest burden was observed at age 8, consistent with findings from European and national datasets, suggesting that mixed dentition remains a vulnerable period for disease progression [42,64].

In the present study, school location and mother’s employment status were significantly associated with the prevalence of untreated decay among children aged 9–11 years. Our results are consistent with the literature data [42,44,65,66,67]. Moreover, a significant relationship between socioeconomic status and oral hygiene has been reported, highlighting the urgent need for oral health promotion initiatives in schools. Although international literature often points to rural populations being more affected [42,57], our data showed that rural children were not disadvantaged compared to their urban peers, as confirmed by dmft/DMFT indices and odds ratios. Our results reflect the influence of socioeconomic status on the oral health of children, because it was shown that the children whose mothers held jobs exhibited lower rates of untreated dental decay and less severe cases of caries compared to their peers whose mothers were unemployed. This correlation may be attributed to the fact that employed mothers often possess higher educational backgrounds, which in turn could enhance their understanding of healthy dental practices. We also found that toothbrushing frequency does not lead to a decrease in tooth decay. This result contradicts the findings of other studies, which reported that a high frequency of toothbrushing leads to a reduction in the prevalence of caries and the risk of their occurrence, and therefore a lower DMFT score [68,69,70,71]. The time of toothbrushing demonstrated a correlation with a reduced incidence of dental caries and less severe carious conditions among children aged 9–11 years within our sample. While the specific toothbrushing time categories did not reach statistical significance among children aged 6–8 years, the overall timing of brushing showed a significant relationship with lower caries prevalence (*p* = 0.04), indicating a possible cumulative effect across all examined categories. Nevertheless, it was observed that fewer than one-third of the children brushed their teeth twice daily, and just over one-third of them brushed for two minutes or longer. The findings presented, although potentially influenced by biases inherent in parental reporting, still suggest that the dental hygiene practices of a significant number of children fall short of the guidelines established by the European Society of Pediatric Dentistry, which advocate for brushing twice daily for two minutes each time with fluoride toothpaste, alongside appropriate oral hygiene education [72]. Once again, the need for oral health educational programs regarding brushing habits was underscored.

In the current investigation, it was found that children aged 6–8 years who had lower sugar intake exhibited a reduced prevalence of untreated dental caries. This finding is consistent with existing literature that indicates a correlation between sugar consumption and the incidence of caries. Specifically, research has shown that children who consume high amounts of sugar, especially before sleeping, face an increased risk of caries development. Furthermore, dietary sugars are identified as a primary risk factor contributing to the onset of carious lesions [73,74,75,76]. We found that the consumption of sugar from soda, natural fruit juices, and sweets did not exhibit a significant correlation with untreated decay in children aged 9–11 years. This outcome contrasts with findings from a previous study involving children of 12 years, which indicated a notable link between the intake of fruit juices and energy drinks and the prevalence of dental caries [77]. As concerns the dental caries experience, only drinking soda several times/week exhibits a significant correlation among children aged 9–11 years. It is very possible that the parents’ reporting of brushing frequency and duration is erroneous, because they were not asked how they controlled and monitored these variables in their children. We believe that this result can be attributed to the maturity of children in this category; older children may be aware of the negative effects of sugar consumption on dental health and take measures to prevent them, without the parents being aware (brush their teeth more often, or after each meal, or use special chewing gum, or certain toothpastes or dental floss); parents were not asked about the latter in the questionnaire. The same explanation can be considered for children’s sugar consumption.

In our research, we observed that children who had not previously visited a dentist exhibited a lower incidence of untreated dental decay compared to those who had sought dental care, whether for routine restorative treatments or emergencies. This observation may indicate a prevalent tendency towards seeking care primarily in response to disease rather than for preventive reasons. Furthermore, this outcome is consistent with earlier investigations conducted in developing nations, which established a correlation between the frequency of dental consults and the elevated rates of dental caries [78,79]. A significant aspect of dental education is its influence on dental service utilization. Children whose parents participate in oral health educational interventions are more likely to visit a dentist regularly. This highlights the correlation between increased knowledge and proactive behaviors. In addition, the authors advocate for early dental consults, as early as the child’s first year of life, suggesting that such initiatives help prevent dental disease, establishing a foundation for continued dental hygiene practices [80].

Our research may have been influenced by social desirability bias, which could lead to an inflated representation of positive oral health practices, such as consistent toothbrushing and minimal sugar intake. To address this issue in subsequent research, incorporating measures that assess social desirability would be beneficial in mitigating such biases. Furthermore, the analysis was based on cross-sectional data, limiting the ability to establish causal relationships. Thus, conducting longitudinal surveys could provide a more accurate understanding of the actual prevalence of the condition in question.

The present study has a few limitations that should be acknowledged. First, its cross-sectional design precludes causal inference, as the observed associations between socioeconomic, behavioral, and dietary factors and caries outcomes may reflect correlation rather than causation. Second, the assessment of behavioral and dietary habits relied on parental self-reported questionnaires, which may be subject to recall bias and social desirability bias. For example, the questions regarding sugar consumption should have been improved with a more detailed range of sugary products, not just those taken into account, as well as the questions regarding oral dental care behaviors: what toothpaste they use, if they use mouthwash or dental floss, etc. Finally, questions regarding the daily diet should have been addressed to the participants, namely the average income per person in a family, how many members the family consists of, the surface area of the house in which they live (number of rooms and bathrooms), etc. Third, although a wide range of covariates were considered, some important potential confounders, such as parental oral health status, household income, and children’s access to dental services, were not available and could not be controlled for in the analyses. These include parental oral health status, household income, and access to dental care services (e.g., geographic proximity to clinics, availability of insurance coverage). Their omission may introduce residual confounding, as these factors are known to influence both health behaviors (e.g., frequency of brushing or dental visits) and oral health outcomes. Future studies should incorporate more detailed socioeconomic profiling and direct measures of parental oral health and access to dental services to more comprehensively assess the determinants of childhood caries. Fourth, the study sample was region-specific and, although relatively large, may not be fully representative of the entire population of Romanian schoolchildren, thus limiting the generalizability of the findings. Finally, while examiner calibration demonstrated excellent reliability, subtle misclassification of caries status cannot be completely excluded, particularly in early-stage lesions.

Another limitation of the study can be attributed to race, ethnicity, or religion; in the present study, we assumed that all the children examined were Romanian, came from Romanian parents, and belonged to the Orthodox religious group. Enhancing understanding regarding the significance of gathering socioeconomic and demographic information can help us to uncover the differences in health outcomes experienced by minority populations. This awareness could lead to a more informed approach to addressing these disparities effectively.

Despite these limitations, the study provides valuable evidence on the prevalence of untreated decay and experience of dental caries in primary schoolchildren and highlights critical socioeconomic and behavioral determinants that may inform future preventive strategies and public health policies.

## 5. Future Directions

The primary recommendation for Romania is to implement targeted oral health strategies aimed at reducing the high prevalence of untreated caries among children. Priority measures include strengthening national school-based oral health programs (regular dental check-ups, fluoride varnish applications, and supervised toothbrushing in schools), expanding access to preventive dental services, especially in rural and underserved areas, and introducing population-wide preventive strategies such as water fluoridation or salt fluoridation where feasible. These measures should be supported by public health campaigns to raise awareness among parents, caregivers, and educators about the importance of diet and oral hygiene.

Building upon the findings and limitations of the present study, several broader directions for future research are warranted. Beyond the national context, these include the need for more comprehensive surveillance of oral health indicators across Europe, cross-country comparative studies to evaluate the effectiveness of public health interventions, and greater integration of oral health into general child health programs. Addressing social inequalities in oral health and ensuring equitable access to preventive care remain central priorities at both national and international levels.

Longitudinal cohort studies are needed to establish temporal and causal relationships between socioeconomic, behavioral, and dietary determinants and the development of dental caries. Such designs would allow for a better understanding of risk accumulation and critical exposure periods in childhood. The inclusion of additional covariates such as parental oral health status, household income, and children’s access to dental care services would provide a more comprehensive picture of the contextual and familial influences on caries risk. More representative, nationally based samples should be investigated to enable generalization of findings to the broader Romanian pediatric population, as well as comparative analyses across regions and socioeconomic groups. Future studies should also explore preventive interventions at both school and family levels, assessing the effectiveness of oral health education programs, dietary counseling, and improved access to routine dental check-ups. Qualitative research involving parents, teachers, and healthcare providers may complement quantitative findings by identifying perceived barriers and facilitators to optimal oral health in children. Taken together, these future directions will help bridge existing knowledge gaps and inform evidence-based policies and targeted public health strategies to reduce the burden of dental caries among school-aged children.

## 6. Conclusions

The study highlights a high prevalence of dental caries among children aged 6–11 in Mureş County, influenced by socioeconomic factors (such as school location and mother’s employment), behavioral habits (like toothbrushing and past dental visits), and dietary choices (consumption of sweets—in the case of children aged 6–8 years and sugary drinks—in the case of children aged 9–11 years). It suggests that public health initiatives should be reevaluated to include more engaging educational programs, using innovative methods to effectively promote lasting oral health behaviors in children.

## Figures and Tables

**Table 1:** Demographic and socioeconomic characteristics of children.

Characteristic	Children Aged 6–8 Years (*N* = 524)	Children Aged 9–11 Years (*N* = 600)
Gender		
- Male	258 (49.2%)	269 (44.8%)
- Female	266 (50.8%)	331 (55.2%)
School location		
- Urban	262 (50%)	276 (46%)
- Rural	262 (50%)	324 (54%)
Mother’s educational level		
- High school	168 (32.1%)	181 (30.2%)
- University	356 (67.9%)	419 (69.8%)
Father’s educational level		
- High school	206 (39.3%)	209 (34.8%)
- University	318 (60.7%)	391 (65.2%)
Mother’s employment status		
- Employed	249 (47.5%)	290 (48.3%)
- Unemployed	275 (52.5%)	310 (51.7%)
Father’s employment status		
- Employed	329 (62.8%)	381 (63.5%)
- Unemployed	195 (37.2%)	219 (36.5%)

**Table 2:** Dental caries among children, by age group, and school location.

Variables	N (%)				Caries Experience ^3^, Mean ± SD	SiC Score ^4^, Mean ± SD
	Decayed ^1^	Missing	Filled	Caries Experience ^2^		
Children aged 6–8 years (*N* = 524)					dmft	dmft
Total	401 (76.5%)	28 (5.3%)	33 (6.3%)	407 (77.7%)	3.9 ± 3.5	7.7 ± 2.5
Urban schools	200 (76.3%)	10 (3.8%)	19 (7.3%)	202 (77.1%)	3.9 ± 3.5	7.6 ± 2.5
Rural schools	201 (76.7%)	18 (6.9%)	14 (5.3%)	205 (78.2%)	3.9 ± 3.5	8.0 ± 2.6
Children aged 9–11 years (*N* = 600)					DMFT	DMFT
Total	261 (43.5%)	41 (7.8%)	38 (6.3%)	289 (48.2%)	1.9 ± 1.8	3.3 ± 3.8
Urban schools	125 (45.3%)	18 (6.5%)	15 (5.4%)	135 (48.9%)	1.6 ± 2.1	3.3 ± 2.0
Rural schools	136 (42.0%)	23 (7.1%)	23 (7.1%)	154 (47.5%)	1.2 ± 1.6	3.2 ± 1.5

^1^ Untreated decay: dt (primary)/DT (permanent) = teeth with untreated carious lesions. ^2^ Caries experience, dmft/DMFT > 0 (including any teeth that are decayed, missing, or filled due to dental decay). ^3^ Mean caries experience: mean dmft (ages 6–8) or mean DMFT (ages 9–11). ^4^ SiC: mean dmft/DMFT of the one-third with the highest dmft/DMFT.

**Table 3:** Children’s distribution related to sugar consumption and oral care.

Characteristic	Children Aged 6–8 Years (*N* = 524)	Children Aged 9–11 Years (*N* = 600)
Sugar consumption		
- Drinking soda	95 (18.1%)	269 (44.8%)
- Drinking natural juice	167 (31.9%)	111 (18.5%)
- Eating sweets	262 (50%)	220 (36.7%)
Toothbrushing habits		
- Brushing frequency	252 (48.1%)	259 (43.2%)
- Brushing time	272 (51.9%)	341 (56.8%)
Dental consult		
- Never	109 (20.8%)	119 (19.8%)
- Treatment	158 (30.2%)	177 (29.5%)
- Emergency	257 (49.0%)	304 (50.7%)

**Table 4:** Multivariable logistic regression models stratified by age group for the prevalence of untreated decay by socioeconomic characteristics, sugar consumption, toothbrushing habits, and dental consult.

	Children Aged 6–8 Years Old				Children Aged 9–11 Years Old			
	Unadjusted		Adjusted		Unadjusted		Adjusted	
	OR (CI 95%)	*p*	OR (CI 95%)	*p*	OR (CI 95%)	*p*	OR (CI 95%)	*p*
Socioeconomic characteristics								
Gender								
Male	1.0		1.0		1.0		1.0	
Female	0.83 (0.62–1.10)	0.18	0.79 (0.52–1.21)	0.26	1.14 (0.88–1.47)	0.42	1.41 (1.01–1.98)	0.08
School location								
Urban	1.0		1.0		1.0		1.0	
Rural	0.99 (0.75–1.35)	0.96	0.53 (0.31–0.92)	0.04	0.82 (0.55–1.22)	0.29	0.50 (0.34–0.75)	<0.001
Mother’s educational level								
High school	1.0		1.0		1.0		1.0	
University	0.81 (0.57–1.17)	0.24	0.93 (0.53–1.65)	0.77	0.90 (0.68–1.19)	0.40	1.15 (0.76–1.74)	0.58
Father’s educational level								
High school	1.0		1.0		1.0		1.0	
University	0.78 (0.57–1.09)	0.14	0.85 (0.51–1.44)	0.53	1.07 (0.82–1.40)	0.74	1.09 (0.73–1.62)	0.77
Mother’s employment status								
Unemployed	1.0		1.0		1.0		1.0	
Employed	1.1 (0.80–1.52)	0.66	0.97 (0.63–1.49)	0.84	0.74 (0.58–0.96)	0.04	0.64 (0.45–0.90)	0.04
Father’s employment status								
Unemployed	1.0		1.0		1.0		1.0	
Employed	0.95 (0.68–1.33)	0.69	0.91 (0.57–1.45)	0.64	1.33 (1.01–1.74)	0.08	1.34 (0.94–1.91)	0.15
Sugar consumption								
Drinking soda		<0.001		0.44		0.88		0.34
Rarely/Never	1.0		1.0		1.0		1.0	
Several times/week	1.81 (1.29–2.55)	<0.001	1.63 (1.0–2.69)	0.35	0.99 (0.72–1.37)	0.89	0.73 (0.49–1.1)	0.13
Once/day	1.95 (1.21–3.17)	0.03	1.45 (0.70–3.04)	0.57	1.28 (0.83–1.96)	0.32	0.82 (0.46–1.46)	0.48
2–3 times/day	1.99 (1.08–3.68)	0.04	1.89 (0.75–4.81)	0.52	1.66 (1.08–2.56)	0.04	1.13 (0.61–2.13)	0.76
≥4 times/day	2.28 (0.67–7.94)	0.22	3.47 (0.30–42.7)	0.66	1.73 (0.53–5.77)	0.41	1.90 (0.28–13.5)	0.55
Drinking natural juice		<0.001		0.24		0.39		0.58
Rarely/Never	1.0		1.0		1.0		1.0	
Several times/week	2.80 (1.81–4.36)	<0.001	2.03 (0.99–4.16)	0.34	1.19 (0.76–1.87)	0.52	1.48 (0.81–2.72)	0.24
Once/day	2.78 (1.72–4.50)	<0.001	1.54 (0.70–3.42)	0.97	1.36 (0.84–2.20)	0.24	1.56 (0.81–3.01)	0.22
2–3 times/day	3.36 (2.03–5.55)	<0.001	1.83 (0.77–4.38)	0.66	1.55 (0.95–2.54)	0.11	1.89 (0.92–3.91)	0.12
≥4 times/day	2.82 (1.25–6.40)	0.03	2.11 (0.51–9.02)	0.84	1.67 (0.67–4.20)	0.32	1.33 (0.42–4.27)	0.67
Eating sweets		<0.001		0.04		0.04		0.35
Rarely/Never	1.0		1.0		1.0		1.0	
Several times/week	2.31 (1.40–3.81)	<0.001	1.85 (0.78–4.43)	0.90	1.01 (0.64–1.60)	0.98	0.79 (0.43–1.48)	0.44
Once/day	2.62 (1.54–4.45)	<0.001	2.73 (1.07–6.97)	0.35	0.93 (0.56–1.55)	0.74	0.70 (0.35–1.41)	0.31
2–3 times/day	3.72 (2.1–6.60)	<0.001	3.51 (1.27–9.72)	0.11	1.31 (0.78–2.21)	0.37	0.87 (0.41–1.86)	0.69
≥4 times/day	2.03 (0.96–4.28)	0.09	1.13 (0.33–3.93)	0.04	2.24 (1.12–4.51)	0.04	1.60 (0.61–4.25)	0.39
Toothbrushing habits								
Frequency of brushing		0.03		0.93		0.39		0.61
Rarely/Never	1.0		1.0		1.0		1.0	
Once/day	0.96 (0.61–1.52)	0.81	1.38 (0.37–5.27)	0.76	0.82 (0.53–1.25)	0.33	0.78 (0.51–1.18)	0.22
2–3 times/day	0.92 (0.58–1.47)	0.69	1.60 (0.42–6.22)	0.54	0.94 (0.61–1.46)	0.74	0.89 (0.57–1.38)	0.55
≥4 times/day	0.41 (0.23–0.77)	0.03	1.89 (0.41–8.97)	0.74	0.78 (0.43–1.42)	0.39	0.73 (0.38–1.45)	0.36
Brushing time		<0.001		0.04		<0.001		<0.001
< 1 min	1.0		1.0		1.0		1.0	
1 min	0.66 (0.42–1.05)	0.09	1.55 (0.85–2.82)	0.12	1.30 (0.87–1.94)	0.25	1.36 (0.85–2.17)	0.25
2 min	0.48 (0.30–0.78)	<0.001	0.98 (0.53–1.83)	0.92	0.83 (0.55–1.26)	0.34	0.78 (0.49–1.27)	0.31
≥3 min	0.44 (0.26–0.74)	<0.001	0.55 (0.27–1.13)	0.12	0.55 (0.31–0.98)	0.04	0.41 (0.21–0.81)	0.03
Dental consult								
Past consult		<0.001		<0.001		<0.001		<0.001
Never	1.0		1.0		1.0		1.0	
Regularly	1.10 (0.73–1.66)	0.75	0.80 (0.41–1.57)	0.50	1.82 (1.17–2.72)	<0.001	1.73 (0.94–3.18)	0.11
Treatment	2.76 (1.62–4.72)	<0.001	3.83 (1.84–7.97)	<0.001	1.96 (1.43–2.69)	<0.001	2.25 (1.51–3.35)	<0.001
Emergency	14.3 (5.17–36.2)	<0.001	9.93 (3.49–25.3)	<0.001	2.37 (1.63–3.45)	<0.001	2.78 (1.77–4.36)	<0.001

OR—odds ratio; CI 95%—confidence interval of 95%; *p*—the significant statistical value (≤0.05).

**Table 5:** Multivariable negative binomial regression models stratified by age group for the dental caries experience by socioeconomic characteristic, sugar consumption, toothbrushing habits, and dental consult.

	Children Aged 6–8 Years Old				Children Aged 9–11 Years Old			
	Unadjusted		Adjusted		Unadjusted		Adjusted	
	RR (CI 95%)	*p*	RR (CI 95%)	*p*	RR (CI 95%)	*p*	RR (CI 95%)	*p*
Socioeconomic characteristics								
Gender								
Male	1.0		1.0		1.0		1.0	
Female	0.95 (0.83–1.09)	0.35	0.96 (0.79–1.16)	0.54	1.02 (0.87–1.23)	0.90	1.18 (0.94–1.48)	0.24
School location								
Urban	1.0		1.0		1.0		1.0	
Rural	0.94 (0.80–1.10)	0.34	1.02 (0.81–1.29)	0.20	0.75 (0.64–0.89)	<0.001	0.61 (0.47–0.81)	<0.001
Mother’s educational level								
High school	1.0		1.0		1.0		1.0	
University	0.88 (0.75–1.05)	1.02	0.86 (0.67–1.10)	0.18	1.09 (0.90–1.31)	0.53	1.07 (0.80–1.42)	0.78
Father’s educational level								
High school	1.0		1.0		1.0		1.0	
University	0.93 (0.80–1.09)	0.14	0.99 (0.79–1.24)	0.79	1.22 (1.01–1.47)	0.06	1.30 (0.98–1.73)	0.12
Mother’s employment status								
Unemployed	1.0		1.0		1.0		1.0	
Employed	1.01 (0.86–1.18)	0.86	1.02 (0.84–1.25)	0.99	0.91 (0.76–1.08)	0.21	0.75 (0.59–0.95)	0.04
Father’s employment status								
Unemployed	1.0		1.0		1.0		1.0	
Employed	1.0 (0.85–1.18)	0.86	1.0 (0.81–1.24)	0.86	1.32 (1.1–1.60)	0.03	1.27 (0.99–1.63)	0.10
Sugar consumption								
Drinking soda		<0.001		0.59		0.14		0.34
Rarely/Never	1.0		1.0		1.0		1.0	
Several times/week	1.46 (1.24–1.72)	<0.001	1.52 (0.60–3.93)	0.43	0.80 (0.65–1.0)	0.04	0.76 (0.57–1.0)	0.04
Once/day	1.27 (1.02–1.58)	0.05	1.05 (0.71–1.56)	0.90	1.01 (0.76–1.33)	0.94	0.78 (0.52–1.17)	0.21
2–3 times/day	1.39 (1.06–1.82)	0.04	1.07 (0.77–1.48)	0.82	1.04 (0.79–1.39)	0.90	0.85 (0.56–1.30)	0.42
≥4 times/day	1.69 (1.02–2.79)	0.05	1.20 (0.97–1.52)	0.15	1.1 (0.51–2.41)	0.87	1.15 (0.34–4.06)	0.87
Drinking natural juice		<0.001		0.24		0.89		0.54
Rarely/Never	1.0		1.0		1.0		1.0	
Several times/week	1.99 (1.55–2.55)	<0.001	1.44 (0.99–2.1)	0.16	0.97 (0.73–1.31)	0.79	0.99 (0.45–2.20)	0.95
Once/day	1.91 (1.46–2.49)	<0.001	1.28 (0.85–1.92)	0.30	0.93 (0.68–1.28)	0.60	1.46 (0.91–2.37)	0.16
2–3 times/day	2.11 (1.62–2.76)	<0.001	1.41 (0.92–2.18)	0.09	1.04 (0.75–1.44)	0.93	1.27 (0.82–1.96)	0.35
≥4 times/day	2.60 (1.75–3.86)	<0.001	1.99 (1.05–3.81)	0.04	0.81 (0.43–1.55)	0.50	1.15 (0.78–1.71)	0.56
Eating sweets		<0.001		0.04		0.80		0.33
Rarely/Never	1.0		1.0		1.0		1.0	
Several times/week	1.95 (1.45–2.61)	<0.001	1.36 (0.84–2.20)	0.26	0.98 (0.73–1.34)	0.84	1.0 (0.53–1.93)	0.98
Once/day	2.11 (1.56–2.85)	<0.001	1.53 (0.93–2.51)	0.13	0.89 (0.64–1.26)	0.50	0.88 (0.53–1.46)	0.59
2–3 times/day	2.36 (1.81–3.34)	<0.001	1.81 (1.08–3.05)	0.04	1.08 (0.76–1.52)	0.78	0.84 (0.52–1.35)	0.44
≥4 times/day	2.24 (1.50–3.35)	<0.001	1.03 (0.54–1.95)	1.01	1.01 (0.63–1.60)	0.98	0.90 (0.60–1.36)	0.58
Toothbrushing habits								
Frequency of brushing		0.03		0.79		0.22		0.53
Rarely/Never	1.0		1.0		1.0		1.0	
Once/day	0.99 (0.80–1.23)	0.76	1.16 (0.64–2.12)	0.70	0.80 (0.59–1.07)	0.12	1.18 (0.75–1.87)	0.55
2–3 times/day	0.98 (0.79–1.23)	0.82	1.02 (0.57–1.85)	1.02	1.21 (0.90–1.62)	0.25	0.98 (0.72–1.33)	0.81
≥4 times/day	0.59 (0.42–0.84)	0.01	1.17 (0.65–2.15)	0.67	1.19 (0.80–1.77)	0.48	0.87 (0.65–1.17)	0.32
Brushing time		0.03		0.23		<0.001		<0.001
< 1 min	1.0		1.0		1.0		1.0	
1 min	0.79 (0.65–0.96)	0.03	0.84 (0.60–1.19)	0.29	1.48 (1.12–1.95)	0.01	1.49(1.07–2.07)	0.03
2 min	0.71 (0.57–0.88)	<0.001	0.83 (0.63–1.10)	0.18	1.11 (0.83–1.49)	0.56	1.09(0.77–1.54)	0.74
≥3 min	0.78 (0.61–1.01)	0.04	1.04 (0.80–1.36)	0.91	0.82 (0.54–1.23)	0.27	0.67(0.41–1.09)	0.08
Dental consult								
Past consult		<0.001		<0.001		<0.001		<0.001
Never	1.0		1.0		1.0		1.0	
Regularly	1.04 (0.84–1.28)	0.89	1.06 (0.75–1.51)	0.85	1.65 (1.22–2.24)	<0.001	1.47 (0.96–2.24)	0.11
Treatment	1.61 (1.31–1.98)	<0.001	1.93 (1.46–2.54)	<0.001	2.12 (1.71–2.64)	<0.001	2.27 (1.72–3.01)	<0.001
Emergency	1.98 (1.62–2.41)	<0.001	2.03 (1.57–2.63)	<0.001	2.15 (1.67–2.77)	<0.001	2.63 (1.93–3.59)	<0.001

RR—rate ratio; CI 95%—confidence interval of 95%; *p*—the significant statistical value (≤0.05).

**Table 6:** Dental health indicators of children according to sugar consumption, oral health behaviors, and past dental consultations of age-specific caries outcomes.

Age of Children (Years)	6	8	11	
Number of children	182	159	218	
	dmft ± SD	dmft ± SD	DMFT ± SD	*p*
Sugar consumption				
Drinking soda	1.95±1.87	1.81±1.71	4.59 ± 4.33	0.04
Drinking natural juice	2.15 ± 2.01	4.33 ± 3.72	1.95 ± 1.59	0.56
Eating sweets	4.05 ± 3.11	2.56 ± 2.67	2.06 ± 1.94	0.03
Oral health behaviors				
Brushing frequency	1.90 ± 1.79	1.85 ± 1.71	2.24 ± 1.91	0.57
Brushing time	3.95 ± 1.18	3.38 ± 2.10	4.10 ± 3.60	<0.001
Dental consult				
Treatment	5.38 ± 2.92	5.20 ± 4.43	4.26 ± 2.93	<0.001
Emergency	6.30 ± 3.38	6.27 ± 3.74	5.31 ± 2.43	<0.001

**Table 7:** Significant Caries Index (SiC) values (mean ± SD) in children aged 6, 8, and 11 years, stratified by dietary habits and oral health behaviours.

Age Children (Years)	6	8	11	
Number of children	182	159	218	
	SiC index ± SD	SiC index ± SD	SiC index ± SD	*p*
Sugar consumption				
Drinking soda	3.85 ± 1.34	3.57 ± 1.22	7.97 ± 9.14	0.04
Drinking natural juice	4.24 ± 1.44	8.55 ± 2.66	3.39 ± 3.36	0.03
Eating sweets	8.00 ± 2.22	5.05 ± 1.91	3.58 ± 4.10	<0.001
Oral health behaviors				
Brushing frequency	3.75 ± 1.28	3.65 ± 1.22	3.89 ± 4.03	0.85
Brushing time	7.80 ± 0.84	6.67 ± 1.50	7.12 ± 7.60	0.53
Dental consult				
Treatment	10.62 ± 2.09	10.27 ± 3.16	7.40 ± 6.19	<0.001
Emergency	12.44 ± 2.41	12.38 ± 2.67	9.22 ± 5.13	<0.001

## Data Availability

All the raw data presented in this study can be provided upon request by the corresponding author.
